# Supplementary material for: Determinants of Podoconiosis in Bensa District, Sidama Region, Ethiopia: A case control study
Source: PLoS Negl Trop Dis. 2023 Aug 29;17(8):e0011502. doi: 10.1371/journal.pntd.0011502 (PMC10464951; doi:10.1371/journal.pntd.0011502)
Supplement: S2 Table — (DOCX) [file pntd.0011502.s002.docx]

Supplement Table 2. Shoe wearing and foot wash practice of participants in Bensa woreda, Sidama Regional state, Ethiopia 2021

| Variables | Category | Case, Frequency (%) | 95% CI | Control, Frequency (%) | 95% CI |
| --- | --- | --- | --- | --- | --- |
| Have you ever wear shoe? | No | 25(16.3) | 11.1, 22.2 | 6(2) | 0.7, 3.9 |
|  | Yes | 128(83.7) | 77.8, 88.9 | 300(98) | 96.1, 99.3 |
| Age at first shoe wear | 2-10 years | 38(29.7) | 18.3, 32 | 214(71.3) | 64.4. 74.8 |
|  | >10 years | 90(70.3) | 51, 66.7 | 86(28.7) | 23.5, 33.3 |
| Wearing status during interview | No | 28(21.9) | 12.4, 24.2 | 22(7.3) | 4.6, 10.1 |
|  | Yes | 100(78.1) | 58.2, 73.2 | 278(92.7) | 87.6. 93.8 |
| Type of shoe | Hard plastic | 27(27) | 23.2, 35.6 | 25(9) | 5.6, 11.1 |
|  | Open sandal | 28(28) | 22, 36.1 | 45(16.2) | 11.1, 19 |
|  | Leather | 15(15) | 6.2, 18 | 129(46.4) | 36.6, 47.7 |
|  | Canvas | 30(30) | 24.3, 40.2 | 79(28.4) | 20.9, 30.7 |
| Time of shoe wearing | At home | 34(26.6) | 15.7, 29.4 | 83(27.7) | 21.9, 32.4 |
|  | During rainy season | 17(13.3) | 6.5, 16.3 | 42(14) | 9.8, 17.6 |
|  | On market day | 37(28.9) | 17, 30.7 | 104(34.7) | 29.1, 39.9 |
|  | On the field | 3(2.3) | 0.0, 4.6 | 6(2) | 0.3, 3.6 |
|  | When walking far | 37(28.9) | 17, 31.4 | 65(21.7) | 16.3, 25.8 |
| Shoe wearing at farm | No | 77(60.2) | 42.5, 57.5 | 122(40.7) | 34.6, 45.8 |
|  | Yes | 51(39.8) | 26.8, 40.5 | 178(59.3) | 52.6, 63.4 |
| Regularly Travel by bare foot | No | 95(74.2) | 58.6, 78.2 | 285(95) | 90.2, 96.1 |
|  | Yes | 33(25.8) | 16.1, 29.5 | 15(5) | 2.6, 7.5 |
| Frequency of wearing shoe | Not daily | 71(55.5) | 40.6, 58.2 | 88(29.3) | 23.9, 33.7 |
|  | Daily | 57(44.5) | 35.5, 50.4 | 212(70.7) | 63.7, 74.2 |
| When do you wash your feet | Whenever they are dirty | 56(36.6) | 28.8, 44.4 | 98(32) | 26.8, 37.3 |
|  | Before sleeping | 83(54.2) | 46.4, 61.4 | 157(51.3) | 45.8, 57.2 |
|  | After completing my job | 14(9.2) | 4.6, 13.7 | 51(16.7) | 12.7, 20.9 |
| Frequency of feet washing | More than once a day | 57(37.3) | 301.1, 45.1 | 102(33.3) | 28.4, 38.9 |
|  | Once a day | 85(55.6) | 48.4, 62.7 | 174(56.9) | 51, 62.1 |
|  | Less often than daily | 9(5.9) | 2.6, 9.8 | 29(9.5) | 6.5, 12.7 |
|  | Weekly or less often | 2(1.3) | 0.0, 3.3 | 1(0.3) | 0.0, 1 |
| Feet washing practice | By water only | 116(75.8) | 68.6, 83 | 173(56.5) | 51, 62.4 |
|  | By water and soap | 37(24.2) | 17, 31.4 | 133(43.5) | 37.6, 49 |
