# Supplementary material for: Determinants of Podoconiosis in Bensa District, Sidama Region, Ethiopia: A case control study
Source: PLoS Negl Trop Dis. 2023 Aug 29;17(8):e0011502. doi: 10.1371/journal.pntd.0011502 (PMC10464951; doi:10.1371/journal.pntd.0011502)
Supplement: Attachment — Submitted filename: PNTD-D-22-01405 comments _OA_June2023.docx [file pntd.0011502.s004.docx]

**Title: Determinants of Podoconiosis in Bensa Woreda, Sidama Region, Ethiopia: A community-based survey**

Manuscript number- PNTD-D-22-01405

**General comment**

It is a well conducted and well reported study.

Most of the comments have been addressed. The remaining comments are stated bellow.

**Comments :**

In Table 2- Type of shoes-canvas among control groups should be 28.4 % ( not 284). The same comment as the previously given one

Line 276 – The Adjusted OR should be 0.07 not 07 (the same comment was given earlier)

Line 339- I think the sentence ‘Finding high odds of podoconiosis among participants with a history of podoconiosis’ , should be stated as ‘Finding high odds of podoconiosis among participants with a **family** history of podoconiosis’
