# Supplementary material for: Determinants of Podoconiosis in Bensa District, Sidama Region, Ethiopia: A case control study
Source: PLoS Negl Trop Dis. 2023 Aug 29;17(8):e0011502. doi: 10.1371/journal.pntd.0011502 (PMC10464951; doi:10.1371/journal.pntd.0011502)
Supplement: Attachment — Submitted filename: Response to reviewers comments.docx [file pntd.0011502.s005.docx]

Many thanks for your resubmission. The manuscript is much improved, but still requires a couple of further changes to fully address the issues raised by Reviewer 1.

Reviewer's Responses to Questions

<b>Key Review Criteria Required for Acceptance?</b></br></br>

As you describe the new analyses required for acceptance, please consider the following:</br></br>

<b>Methods</b></br></br>

-Are the objectives of the study clearly articulated with a clear testable hypothesis stated?</br>

-Is the study design appropriate to address the stated objectives?</br>

-Is the population clearly described and appropriate for the hypothesis being tested?</br>

-Is the sample size sufficient to ensure adequate power to address the hypothesis being tested?</br>

-Were correct statistical analysis used to support conclusions?</br>

-Are there concerns about ethical or regulatory requirements being met?</br></br>

Reviewer #1: This submission is a revised version of PNTD-D-22-01405R1. The authors have provided comprehensive responses to the comments raised.

**Response:** Thank you

Reviewer #2: The objectives of the study are clearly articulated with a clear testable hypothesis. The study design is appropriate to address these objectives. The population is clearly described and it is appropriate for the hypothesis being tested. The sample size is sufficient to ensure adequate power to address the hypothesis being tested. Correct statistical analysis has been used to support conclusions. The ethical issues are well addressed.

**Response:** Thank you

--------------------

<b>Results</b></br></br>

-Does the analysis presented match the analysis plan?</br>

-Are the results clearly and completely presented?</br>

-Are the figures (Tables, Images) of sufficient quality for clarity?</br></br>

Reviewer #1: Yes

Reviewer #2: The analysis presented matches the analysis plan.

The results are clearly and completely presented. The way the results organized are well detailed and well organized.

The statistical analysis results are well interpreted.

The figures and tables are good quality and clarity.

**Response:** Thank you

--------------------

<b>Conclusions</b></br></br>

-Are the conclusions supported by the data presented?</br>

-Are the limitations of analysis clearly described?</br>

-Do the authors discuss how these data can be helpful to advance our understanding of the topic under study?</br>

-Is public health relevance addressed?</br></br>

Reviewer #1: The authors chose not to reanalyse their data to correct for the biased age distribution of controls, but have provided some explanation in the limitations section. I am still concerned that the sampling of controls has biased the estimate of the effect of age (the true odds ratio for podo in females versus males likely to be closer to 1.5 than 3). This is of concern because the presented AOR of 3 may be quoted in the future or included in systematic reviews/ meta-analyses without consideration of the impact of the sampling bias. With this in mind, I think the authors should be more transparent in the limitations section about how this bias is expected to have affected the results (beyond saying it “can bias the finding of this study”). For example- “we acknowledge that the preponderance of males in the control sample is likely to have led us to overestimate the association of podoconiosis with gender”.

The limitations section also states that the authors used systematic random sampling. Systematic random sampling is intended to select sample data that represents a population. However, the systematic random sampling in this study was not applied to the study population, but to the kebele register, which was recognised to be biased as females were much less likely to be registered. I would therefore suggest removing “Even though we have employed systematic random sampling”, as some readers may interpret this as systematic random sampling of the population.

**Response:** Thank you so much for the comment; we have included the information suggested and removed a statement ‘Even though we have employed systematic random sampling’ from the manuscript. The change is shown in the manuscript with track change.

Reviewer #2: The conclusions are  supported by the data presented.

The limitations of analysis are clearly described. The public health relevance including risk factors and potential prevention mechanisms has been addressed.

The authors discuss how these data can be helpful to advance our understanding of Podoconiosis.

**Response**: Thank you

--------------------

<b>Editorial and Data Presentation Modifications?</b></br><br/>

Use  this section for editorial suggestions as well as relatively minor modifications of existing data that would enhance clarity. If the only modifications needed are minor and/or editorial, you may wish to recommend “Minor Revision” or “Accept”.

Reviewer #1: “The geographic information was downloaded to the computer program to generate a spatial 161 distribution of cases.” – this can be removed as the geographic mapping method is described fully later on.

**Response:** Thank you very much for the comment we have omitted the statement.

“Finding high odds of podoconiosis among participants with a history of podoconiosis” – a family history

“waking barefoot” (page 20 and 28) – walking

**Response:** Thank you for pointing this error, we have corrected it.

Reviewer #2: “Accept” with minor edits as shown bellow (It is also attached)

In Table 2- Type of shoes-canvas among control groups should be 28.4 % ( not 284). The same comment as the previously given one

**Response**: Thank you very much for pointing the error, we have corrected it.

Line 276 – The Adjusted OR should be 0.07 not 07 (the same comment was given earlier)

**Response**: Thank you very much for pointing the error, we have corrected it.

Line 339- I think the sentence ‘Finding high odds of podoconiosis among participants with a history of podoconiosis’ , should be stated as ‘Finding high odds of podoconiosis among participants with a  family history of podoconiosis’

**Response**: Thank you very much for pointing this, we have corrected it.

**Response for separate comment attached**

**Title: Determinants of Podoconiosis in Bensa Woreda, Sidama Region, Ethiopia: A community-based survey**

Manuscript number- PNTD-D-22-01405

**General comment**

It is a well conducted and well reported study.

Most of the comments have been addressed. The remaining comments are stated bellow.

**Comments :**

In Table 2- Type of shoes-canvas among control groups should be 28.4 % ( not 284). The same comment as the previously given one.

**Response**: Thank you very much for pointing the error, we have corrected it.

Line 276 – The Adjusted OR should be 0.07 not 07 (the same comment was given earlier)

Line 339- I think the sentence ‘Finding high odds of podoconiosis among participants with a history of podoconiosis’ , should be stated as ‘Finding high odds of podoconiosis among participants with a **family** history of podoconiosis’

**Response**: Thank you very much for pointing this, we have corrected it.

--------------------

<b>Summary and General Comments</b></br></br>

Use  this section to provide overall comments, discuss strengths/weaknesses of the study, novelty, significance, general execution and scholarship. You may also include additional comments for the author, including concerns about dual publication, research ethics, or publication ethics. If requesting major revision, please articulate the new experiments that are needed.

Reviewer #1: (No Response)

Reviewer #2: It is a well conducted and well reported study.

Most of the comments have been well addressed.

Though the topic is not novel , there was no similar study in this study site. The determination of risk factors will help in the prevention and control of podoconiosis.

It would have been better if this case control study was properly matched.

Response: Thank you

--------------------

PLOS authors have the option to publish the peer review history of their article (<a href="https://journals.plos.org/plosntds/s/editorial-and-peer-review-process#loc-peer-review-history" target="_blank">what does this mean?</a>). If published, this will include your full peer review and any attached files.<br><br>

If you choose “no”, your identity will remain anonymous but your review may still be made public.<br><br>

<b>Do you want your identity to be public for this peer review?</b> For information about this choice, including consent withdrawal, please see our <a href="https://www.plos.org/privacy-policy" target="_blank">Privacy Policy</a>.

Reviewer #1: No

Reviewer #2: No

Figure Files:

While revising your submission, please upload your figure files to the Preflight Analysis and Conversion Engine (PACE) digital diagnostic tool, https://pacev2.apexcovantage.com. PACE helps ensure that figures meet PLOS requirements. To use PACE, you must first register as a user. Then, login and navigate to the UPLOAD tab, where you will find detailed instructions on how to use the tool. If you encounter any issues or have any questions when using PACE, please email us at [figures@plos.org.](mailto:figures@plos.org.)

Data Requirements:

Please note that, as a condition of publication, PLOS' data policy requires that you make available all data used to draw the conclusions outlined in your manuscript. Data must be deposited in an appropriate repository, included within the body of the manuscript, or uploaded as supporting information. This includes all numerical values that were used to generate graphs, histograms etc.. For an example see here: http://www.plosbiology.org/article/info%3Adoi%2F10.1371%2Fjournal.pbio.1001908#s5.

Reproducibility:

To enhance the reproducibility of your results, we recommend that you deposit your laboratory protocols in protocols.io, where a protocol can be assigned its own identifier (DOI) such that it can be cited independently in the future. Additionally, PLOS ONE offers an option to publish peer-reviewed clinical study protocols. Read more information on sharing protocols at <https://plos.org/protocols?utm_medium=editorial-email&utm_source=authorletters&utm_campaign=protocols>

References

Please review your reference list to ensure that it is complete and correct. If you have cited papers that have been retracted, please include the rationale for doing so in the manuscript text, or remove these references and replace them with relevant current references. Any changes to the reference list should be mentioned in the rebuttal letter that accompanies your revised manuscript. If you need to cite a retracted article, indicate the article's retracted status in the References list and also include a citation and full reference for the retraction notice.

__________________________________________________

In compliance with data protection regulations, you may request that we remove your personal registration details at any time.  (Use the following URL: https://www.editorialmanager.com/pntd/login.asp?a=r). Please contact the publication office if you have any questions.
